# Stage-specific risks of mortality and renal outcomes in cardiovascular-kidney-metabolic syndrome: findings from a nationwide Japanese cohort

**DOI:** 10.1007/s10157-025-02800-x

**Published:** 2025-12-17

**Authors:** Kenta Fujimoto, Masao Kikuchi, Michikazu Nakai, Tsuneo Konta, Kunitoshi Iseki, Kazuhiko Tsuruya, Kunihiro Yamagata, Ichiei Narita, Toshiki Moriyama, Yugo Shibagaki, Masato Kasahara, Masahide Kondo, Koichi Asahi, Tsuyoshi Watanabe, Koichi Kaikita, Shouichi Fujimoto

**Affiliations:** 1 Division of Cardiovascular Medicine and Nephrology, Department of Internal Medicine, Faculty of Medicine, University of Miyazaki, Miyazaki, Japan; 2 Department of Statistics and Data Management, Faculty of Medicine, University of Miyazaki, Miyazaki, Japan; 3 Steering Committee of The Japan Specific Health Checkups (J-SHC) Study, Fukushima, Japan; 4 M&N Collaboration Research Laboratory, Department of Medical Environment Innovation, Faculty of Medicine, University of Miyazaki, Miyazaki, Japan

**Keywords:** Cardiovascular disease, Cardiovascular-kidney-metabolic syndrome, Chronic kidney disease, Metabolic dysfunction, Mortality

## Abstract

**Background:**

Cardiovascular-kidney-metabolic (CKM) syndrome, integrating cardiovascular disease (CVD), chronic kidney disease (CKD), and metabolic dysfunction, is a construct proposed by the American heart association. Although associations with CVD are well recognized, evidence linking CKM stage to renal outcomes remains limited.

**Methods:**

We analyzed health checkup data of 266,256 Japanese aged 40–74 years. Participants were classified into CKM stages 0–4a. Outcomes included all-cause mortality, cardiovascular death, and a composite renal outcome (end-stage kidney disease [eGFR < 15 mL/min/1.73 m^2^], ≥ 40% eGFR decline, or doubling of serum creatinine). Multivariable Cox proportional hazards models were used to estimate hazard ratios (HRs), with CKM stage 0 as the reference.

**Results:**

CKM stage 2 was the most prevalent stage (65.0%). Stage 4a showed the strongest association with all-cause and cardiovascular mortality (HRs 1.79, 3.16; 95% CIs 1.41–2.28, 1.92–5.20, respectively). In contrast, stage 3 conferred the highest risk of renal outcomes (HR 15.29, 95% CI 10.13–23.08). The number and type of metabolic risk factors correlated with outcomes, furthermore, severe CKD and prior CVD were stronger drivers of adverse outcomes than metabolic dysfunction.

**Conclusion:**

CKM staging stratifies risk in the general population. No significant increase in risk was observed until CKM stage 2, and these findings underscore the progressive, cumulative nature of CKM syndrome. Metabolic dysfunction plays a crucial role in progression, stage 3 marks a pivotal inflection point for renal deterioration, and stage 4a identifies individuals at the greatest mortality risk. Early interventions targeting metabolic dysfunction may help prevent progression to advanced CKM stages and improve long-term outcomes.

**Supplementary Information:**

The online version contains supplementary material available at 10.1007/s10157-025-02800-x.

## Introduction

Cardiovascular-kidney-metabolic (CKM) health is important for reducing mortality and several serious complications. The American heart association (AHA) has proposed a group of diseases as CKM syndrome [1]. CKM syndrome is an integrated disease construct encompassing cardiovascular disease (CVD), chronic kidney disease (CKD), and metabolic factors [1]. The AHA recommends screening for CKM syndrome in populations over 30 years of age and assessing the risk of CVD [2]. A worsening CKM health status is a major determinant of early complications and mortality [1].

Many of the factors associated with CKM syndrome are well-known risk factors for chronic and end-stage kidney failure. Hypertension [3], diabetes [4, 5], obesity [6, 7], all of which have been identified as risk factors for kidney damage in multiple previous studies, and correction of these factors is a component of treatment. Many studies have shown a strong association between CKD and CVD, and that metabolic dysfunction is an exacerbating factor in both conditions. On the other hand, there is little evidence on renal disease outcomes in CKM syndrome, especially in Asian populations, including the Japanese population, which has not been evaluated to date.

We speculate that it is important to appropriately assess the risk of CKD progression in each CKM syndrome category and consider countermeasures and treatments. This study describes the differences in risk assessment, all-cause mortality, cardiovascular mortality, and renal composite outcomes for each stage of CKM syndrome using health examination data from a Japanese population, which may assist in determining appropriate treatment strategies.

## Methods

### Study design and participants

This longitudinal cohort study was based on the Japan specific health checkups (J-SHC) study, a Japanese general population health checkup project. The Specific health checkups program is conducted annually. Accordingly, for each participant we defined the earliest available examination as baseline and the latest as follow-up. Between 2008 and 2014, a total of 423,009 individuals aged 40–74 years participated in this study. The details of this study are described elsewhere [8].

**Fig. 1:**
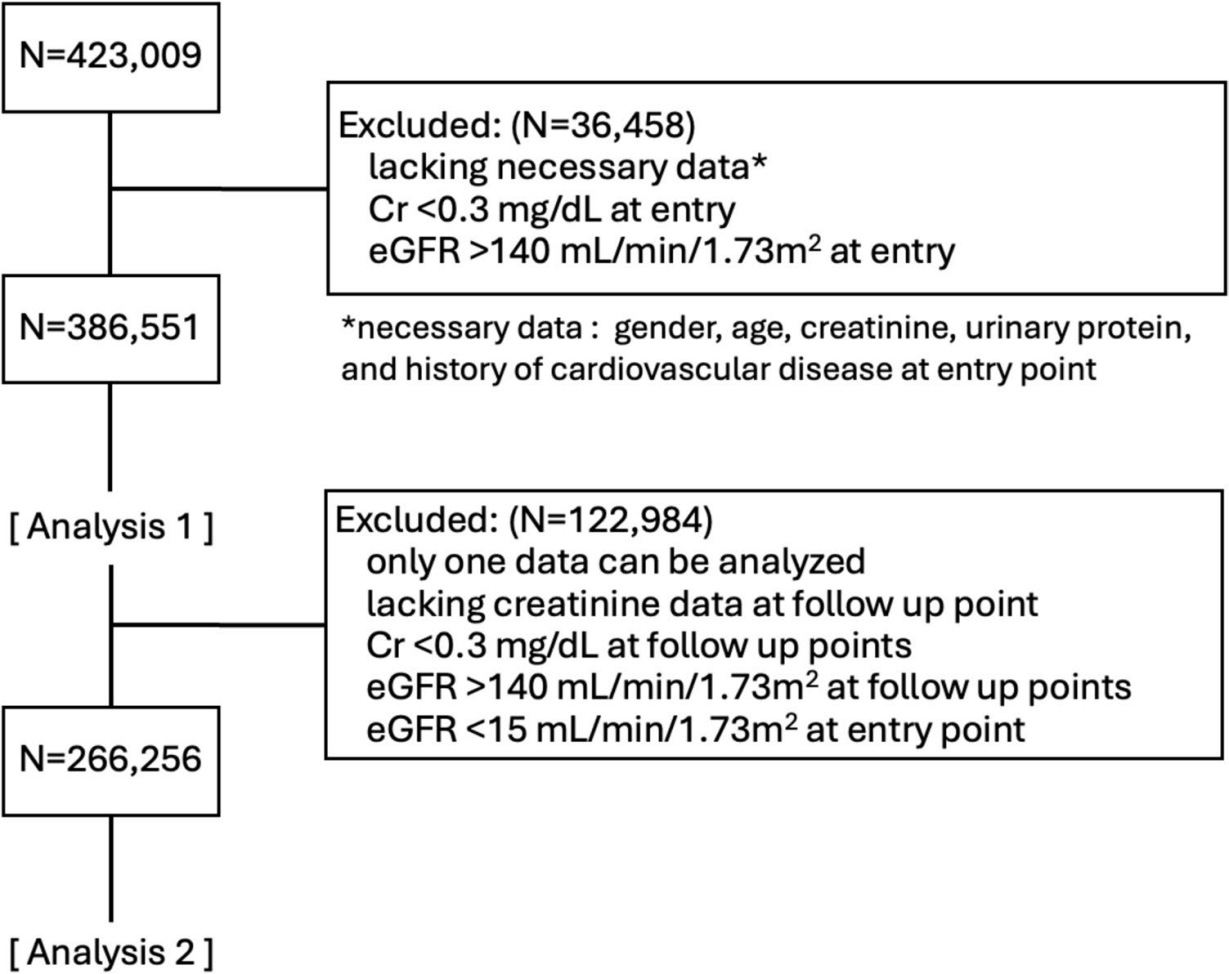
Flowchart of the study participants. This figure shows the selection process for the study population

Participants with missing key baseline data, including sex, age, serum creatinine level, urinary protein level, and CVD history. Or implausible creatinine/eGFR values (creatinine < 0.3 mg/dL or eGFR > 140 mL/min/1.73 m^2^) were excluded. After these exclusions, 386,551 participants were included in analysis 1. For longitudinal analysis, we further excluded those with only one time point of data or those with missing follow-up creatinine, or eGFR < 15 mL/min/1.73 m^2^ at baseline were excluded. Ultimately, 266,256 participants were included in analysis 2 (Fig. 1).

To ascertain mortality outcomes, approval was obtained from the ministry of health, labour and welfare, and participants were linked to the national death certificate registry. Potential matches were verified in collaboration with local national health insurance associations and public health nurses. All personal identifiers were fully encrypted under strict government regulations. Causes of death were coded according to the international classification of diseases, 10th revision (ICD-10), and cardiovascular mortality was defined using ICD-10 codes beginning with “I”. All analyses used a standardized, anonymized dataset (standard analysis file version 3.5).

Ethical approval was obtained from Fukushima medical university (#1485, #2771), the university of Tsukuba (#999), and the university of Miyazaki (IRB #O-0117). In accordance with national guidelines, information was publicly disclosed and participants were provided the opportunity to opt out; individual consent was not required. All data were anonymized before analysis.

### Data collection

All data were obtained during the fasting period. Blood pressure (BP) was measured after a 5 min rest. Proteinuria was assessed using dipstick testing (− to ≥ 3 +), categorized as A1 (−), A2 (±), and A3 (≥ 1 +), following the Japanese CKD guidelines 2023 [9]. Serum creatinine levels were measured using an enzymatic method. eGFR is defined by the Japanese GFR equation [10]. CKD was defined as an eGFR < 60 mL/min/1.73 m^2^ or proteinuria classified as A2 or A3. Because the health checkups were conducted annually, CKD classification relied on a single measurement, rather than the standard requirement of persistent abnormalities; this limitation was acknowledged. Diabetes was defined according to the American diabetes association guidelines [11] as HbA1c ≥ 6.5%, fasting plasma glucose ≥ 126 mg/dL, or the use of diabetes medications. Hypertension was defined according to the JSH 2019 guidelines [12] as systolic BP ≥ 130 mmHg, diastolic BP ≥ 80 mmHg, or the use of antihypertensive medications. Data on each medication and social and past medical history were confirmed using a questionnaire survey. CVD history was self-reported and included heart disease and cerebrovascular disease; data on peripheral arterial disease and atrial fibrillation were unavailable.

### Definition of CKM stage and outcomes

Baseline CKM syndrome was classified into six categories based on criteria proposed by the AHA [1], incorporating CKD severity, comorbidities, body mass index (BMI), and laboratory data. Patients without any apparent cardiovascular, renal, or metabolic risk factors were classified as stage 0. Those with obesity or prediabetes but no overt disease were classified as stage 1. Patients with established metabolic abnormalities and/or CKD were assigned to stage 2. Those with criteria for very high-risk CKD (severe CKD) according to the KDIGO criteria [13] or with an estimated 10-year risk of developing heart disease of 20% or greater using the predicting risk of CVD EVENTs (PREVENT) equation [14] were classified as stage 3. However, the lack of data on clinical parameters such as B-type natriuretic peptide (BNP), N-terminal proBNP (NT-proBNP), high-sensitivity cardiac troponins, and cardiac imaging could have led to an underestimation of stage 3 classification. Stage 4a included patients with a history of CVD without kidney failure. Kidney failure is defined as eGFR < 15 mL/min/1.73 m^2^ in the KDIGO guideline [15]. Stage 4b included patients with both history of CVD and kidney failure. Because individuals with baseline eGFR < 15 mL/min/1.73 m^2^ were excluded, all participants categorized as stage 4 in the present analysis correspond to stage 4a in analysis 2, regardless of CKD status. The detailed criteria used for the CKM stage classification in this study are summarized in Table 1.

**Table 1:** Determination of cardiovascular-kidney-metabolic (CKM) staging in this study

CKM stage	NHANES mapping/description
Stage 0	No CKM health risk factors
	No criteria met for other stages
Stage 1	Excess and/or dysfunctional adiposity
	a. Elevated BMI ≥ 23 kg/m^2^
	b. Prediabetes (HbA1c 5.7–6.4% or fasting glucose 100–125 mg/dL)
	c. Waist circumference ≥ 80/90 cm in women/men
	Condition: No chronic kidney disease (CKD) and no cardiovascular disease (CVD), with at least one of a–c
Stage 2	Metabolic risk factors and CKD
	a. Diabetes (HbA1c ≥ 6.5%, fasting glucose ≥ 126 mg/dL, or self-reported diabetes)
	b. Hypertriglyceridemia [≥ 135 mg/dL]
	c. Hypertension (systolic blood pressure ≥ 130 mm Hg or diastolic blood pressure ≥ 80 mm Hg and/or use of antihypertensive medications)
	d. Metabolic syndrome (≥ 3 of the following)
	(1) Waist circumference ≥ 80 cm for women and ≥ 90 cm for men
	(2) HDL cholesterol < 40 mg/dL for men and < 50 mg/dL for women
	(3) Triglycerides ≥ 150 mg/dL
	(4) Elevated blood pressure (systolic blood pressure ≥ 130 mm Hg or diastolic blood pressure ≥ 80 mm Hg and/or use of antihypertensive medications)
	(5) Fasting blood glucose ≥ 100 mg/dL
	e. CKD (eGFR < 60 mL/min/1.73 m^2^ or urinary protein A2 or A3)
	Condition: No CVD, with at least one of a–e
Stage 3	Subclinical CVD and CKD
	a. Very-high-risk CKD (per KDIGO criteria)
	b. High predicted 10-y CVD risk (PREVENT equation score ≥ 20%)
	Condition: No CVD, with at least one of a or b
Stage 4a	Clinical CVD without kidney failure
	Individuals with history of CVD without kidney failure (defined as eGFR ≥ 15 mL/min/1.73 m^2)^
Stage 4b	Clinical CVD with kidney failure
	Individuals with history of CVD with kidney failure (defined as eGFR < 15 mL/min/1.73 m^2^)

This table summarizes the criteria used to classify CKM stages 0–4b in this study. Cardiac biomarkers and imaging parameters were not available and were therefore not included in the definition of stage 3. In analysis 1, both stage 4a and stage 4b were present. In analysis 2, individuals who met the criterion for kidney failure (eGFR < 15 mL/min/1.73 m^2^), corresponding to stage 4b, were excluded. Consequently, in analysis 2, all participants with a history of cardiovascular disease (CVD) were classified as stage 4a regardless of kidney function

A renal composite outcome was defined as a decline in eGFR of 40% or more, doubling of serum creatinine, or progression to end-stage kidney disease (ESKD), which was defined as progression to eGFR < 15 mL/min/1.73 m^2^ in the follow-up data.

### Statistical analysis

Continuous variables were summarized as medians with interquartile ranges (IQRs) and categorical variables as frequencies and percentages. Associations between CKM stage and outcomes were evaluated using multivariable Cox proportional hazards models adjusted for age, sex, smoking, alcohol consumption, and exercise habits, with stage 0 as reference. Hazard ratios (HRs) with 95% confidence intervals were reported. All analyses were performed using JMP^®^ student edition version 18.2.0 (SAS Institute Inc., Cary, NC, USA). A two-sided P value < 0.05 was considered statistically significant.

## Results

### Prevalence of CKM stages

**Fig. 2:**
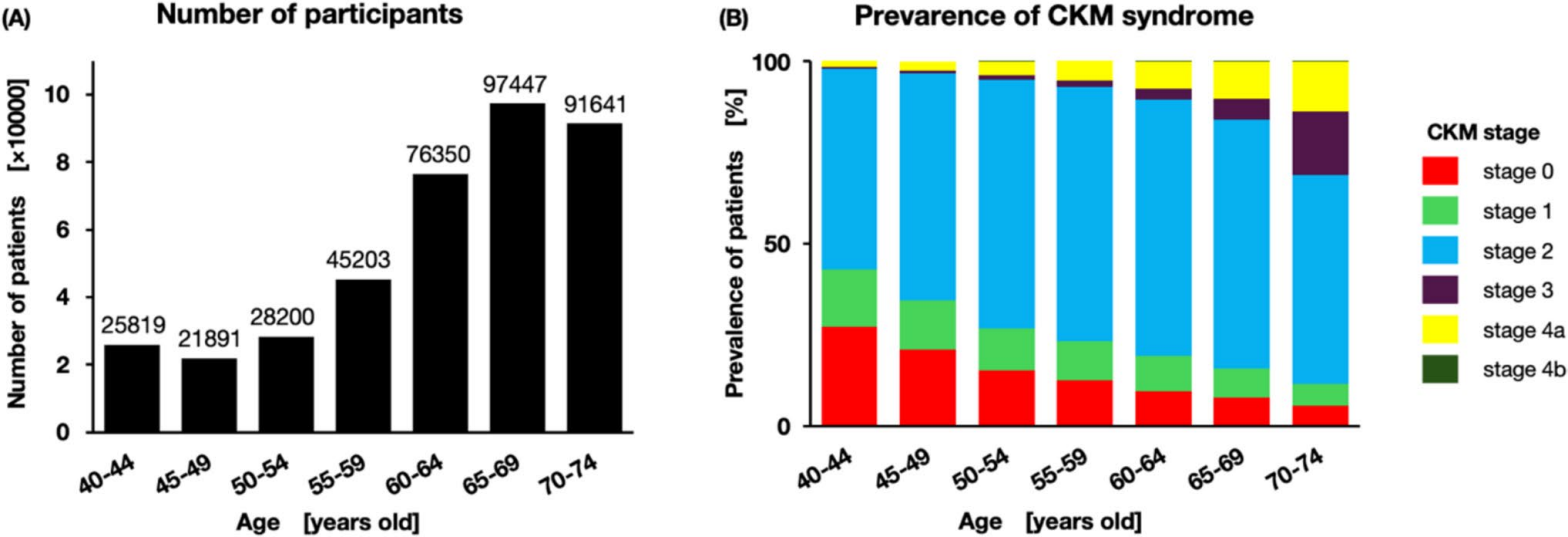
Age-stratified participants (**A**) and prevalence of each CKM stage (**B**) in Japan (analysis 1). Analysis 1 consisted of a cross-sectional study of a total of 386,551 participants. Panel A shows the number of participants by 5-year age groups. Panel B shows the prevalence of CKM stages 0–4b within each age group. Stage 2 was the most prevalent across all ages, while the prevalence of higher stages increased with age. Stage 2 was the most prevalent in all age groups. The higher stages, particularly stages 3–4, became increasingly common with increasing age. The proportions of stage 0 and stage 1 decreased with age, whereas stage 3 and above increased sharply from the 60 s onward

Figure 2 presents the age-stratified prevalence of CKM stages (analysis 1). A total of 386,551 individuals were included in analysis 1. The prevalence of each stage was as follows: stage 0, 10.71%; stage 1, 9.13%; stage 2, 65.04%; stage 3, 6.57%; stage 4a, 8.54%; stage 4b, 0.02% (Fig. 2 and Supplemental Table 1). Stage 2 was the most frequent across all age groups (55–70%). Younger participants were more likely to be classified as stage 0 or 1, but this proportion decreased with age, to just 11% of those older than 70 years. In contrast, the prevalence of stages 3–4 increased steadily with age, from 2.1% in the youngest group to 31% in the oldest. Stage 3, in particular, rose sharply in older participants, largely driven by the higher proportion with PREVENT equation scores ≥ 20.

### Baseline characteristics and prevalence of CKM stages in analysis 2

Table 2 summarizes the baseline characteristics of the participants across CKM stages (analysis 2). Stage 0 represents the general population without risk factors, whereas stage 1 comprises individuals with fat accumulation and/or glucose intolerance during the pre-disease stage. Although BMI differed markedly between the two groups, other laboratory parameters were largely comparable. Owing to the higher female participants, hemoglobin levels were lower in stages 0 and 1 than in the other stages. Notably, the proportion of participants using lipid-lowering agents, although not included in the CKM staging criteria, increased progressively with advancing stage from 5.06% in stage 0 to 28.01% in stage 4 (Table 2).

**Table 2:** Comparison of characteristics by CKM stage at baseline in the analyzed population (analysis 2)

CKM stage	Stage 0	Stage 1	Stage 2	Stage 3	Stage 4a	All
Number, N (%)	29,262 (10.99%)	25,441 (9.56%)	175,589 (65.95%)	14,213 (5.34%)	21,751 (8.17%)	266,256 (100.00%)
Sex (female), N (%)	20,842 (71.23%)	19,300 (75.86%)	101,940 (58.06%)	3380 (23.78%)	10,253 (47.14%)	155,715 (58.48%)
Age, years	60 (51–66)	62 (55–67)	65 (59–69)	70 (67–72)	67 (63–71)	65 (58–69)
Height, cm	156 (151.1–162)	154.6 (150–160.4)	156.3 (150.5–163.5)	160.5 (154.6–165.3)	157.5 (151.2–164)	156.4 (150.7–163.2)
Weight, kg	48.7 (44.5–53.8)	56.3 (51.5–63.1)	57.8 (51–65.5)	63 (56.6–69.8)	59 (52–66.3)	56.9 (50.2–64.7)
BMI, kg/m^2^	20.2 (18.9–21.4)	23.6 (22.2–25.1)	23.5 (21.5–25.7)	24.5 (22.6–26.8)	23.7 (21.7–25.85)	23.2 (21.1–25.4)
SBP, mmHg	111 (104–120)	115 (108–121)	132 (122–141)	140 (130–152)	130 (120–140)	128 (118–140)
DBP, mmHg	68 (60–71)	70 (63–72)	80 (71–85)	80 (72–87)	76 (70–82)	76 (70–82)
Hemoglobin, g/dL	13.0 (12.2–13.8)	13.1 (12.4–13.9)	13.6 (12.8–14.6)	14.2 (13.1–15.25)	13.7 (12.8–14.7)	13.5 (12.7–14.5)
Serum creatinine, mg/dL	0.6 (0.6–0.7)	0.6(0.6–0.7)	0.7 (0.6–0.8)	0.9 (0.8–1.1)	0.8 (0.6–0.9)	0.7 (0.6–0.8)
eGFR, mL/min/1.73 m^2^	76.69 (69.41–86.97)	75.75 (67.41–86.35)	74.09 (63.91–83.99)	58.29 (51.20–72.86)	70.04 (61.58–77.06)	74.06 (63.91–83.99)
Fasting glucose, mg/dL	89 (84–94)	91 (86–97)	94 (88–102)	104 (93–129)	95 (88–105)	93 (87–101)
HbA1c, %	5.1 (4.9–5.3)	5.2 (5–5.4)	5.2 (5–5.5)	5.6 (5.2–6.5)	5.3 (5–5.6)	5.2 (5–5.5)
Triglycerides, mg/dL	73 (57–93)	85 (66–106)	111 (78–159)	126 (90–181)	105 (76–148)	101 (73–145)
HDL-C, mg/dL	69 (59–80)	63 (54–73)	59 (49–70)	50 (43–59.175)	56 (47–67)	60 (50–71)
LDL-C, mg/dL	118 (99–137)	127 (108–147)	127 (107–148)	121 (102–141)	119 (100–139)	125 (105–145)
Total cholesterol, mg/dL	203.2 (181.8–225.2)	208.6 (187.6–231)	213.4 (191.4–237)	202.2 (180.2–226)	202.4 (180.6–224.8)	210.4 (188.4–233.6)
Uric acid, mg/dL	4.5 (3.8–5.2)	4.7 (4.1–5.5)	5.3 (4.4–6.2)	6.1 (5.1–7)	5.5 (4.6–6.5)	5.2 (4.3–6.2)
PREVENT equation score, %	4.11 (1.84–7.18)	4.96 (2.78–7.73)	9.02 (5.53–12.85)	22.00 (20.43–24.77)	12.29 (8.08–16.82)	8.59 (4.84–13.14)
Follow-up period, years	4.56 (3.31–5.01)	4.54 (3.29–5.01)	4.30 (3.12–4.99)	3.25 (2.28–4.53)	3.93 (2.89–4.95)	4.23 (3.08–4.99)
Urinary protein						
(−)	29,262 (100.00%)	25,441 (100.00%)	148,993 (84.85%)	8289 (58.32%)	17,905 (82.32%)	229,890 (86.34%)
(±)	0 (0.00%)	0 (0.00%)	18,192 (10.36%)	1531 (10.77%)	1929 (8.87%)	21,652 (8.13%)
(1 +)	0 (0.00%)	0 (0.00%)	6324 (3.60%)	2596 (18.26%)	1214 (5.58%)	10,134 (3.81%)
(2 +)	0 (0.00%)	0 (0.00%)	1656 (0.94%)	1347 (9.48%)	530 (2.44%)	3533 (1.33%)
(3 +)	0 (0.00%)	0 (0.00%)	424 (0.24%)	450 (3.17%)	173 (0.80%)	1047 (0.39%)
Taking hypertension medicine						
Yes	0 (0.00%)	0 (0.00%)	52,061 (29.65%)	9782 (68.82%)	12,480 (57.38%)	74,323 (27.91%)
No	29,262 (100.00%)	25,441 (100.00%)	123,527 (70.35%)	4431 (31.18%)	9271 (42.62%)	191,932 (72.09%)
Taking diabetes medicine						
Yes	0 (0.00%)	0 (0.00%)	7411 (4.22%)	3738 (26.30%)	2193 (10.08%)	13,342 (5.01%)
No	29,262 (100.00%)	25,441 (100.00%)	168,178 (95.78%)	10,475 (73.70%)	19,558 (89.92%)	252,914 (94.99%)
Taking dyslipidemia medicine						
Yes	1482 (5.06%)	2142 (8.42%)	24,150 (13.75%)	2433 (17.12%)	6092 (28.01%)	36,299 (13.63%)
No	27,780 (94.94%)	23,299 (91.58%)	151,439 (86.25%)	11,780 (82.88%)	15,659 (71.99%)	229,957 (86.37%)
Smoking status						
Yes	4145 (14.17%)	2532 (9.95%)	22,431 (12.77%)	4210 (29.62%)	2353 (10.82%)	35,671 (13.40%)
No	25,117 (85.83%)	22,909 (90.05%)	153,158 (87.23%)	10,003 (70.38%)	19,398 (89.18%)	230,585 (86.60%)
Exercise status						
Yes	10,128 (38.82%)	9,164 (41.00%)	68,487 (45.09%)	6465 (53.39%)	9537 (48.86%)	103,781 (44.74%)
No	15,962 (61.18%)	13,187 (59.00%)	83,388 (54.91%)	5645 (46.61%)	9982 (51.14%)	128,164 (55.26%)
Habit of alcohol						
Yes (everyday)	5138 (18.00%)	3392 (13.72%)	39,150 (22.87%)	4427 (31.91%)	4862 (22.89%)	56,969 (21.95%)
No (or sometimes)	23,399 (82.00%)	21,330 (86.28%)	132,014 (77.13%)	9446 (68.09%)	16,379 (77.11%)	202,568 (78.05%)
History of CVD						
Yes	0 (0.00%)	0 (0.00%)	0 (0.00%)	0 (0.00%)	21,751 (100.00%)	21,751 (8.17%)
No	29,262 (100.00%)	25,441 (100.00%)	175,589 (100.00%)	14,213 (100.00%)	0 (0.00%)	244,505 (91.83%)
History of CKD						
Yes	0 (0.00%)	0 (0.00%)	51,573 (29.37%)	9251 (65.09%)	7737 (35.57%)	68,561 (25.75%)
No	29,262 (100.00%)	25,441 (100.00%)	124,016 (70.63%)	4962 (34.91%)	14,014 (64.43%)	197,695 (74.25%)
History of diabetes						
Yes	0 (0.00%)	0 (0.00%)	9439 (5.42%)	4817 (33.96%)	2478 (11.46%)	16,734 (6.32%)
No	29,261 (100.00%)	25,440 (100.00%)	164,784 (94.58%)	9367 (66.04%)	19,141 (88.54%)	247,993 (93.68%)

Data are presented as n (%) or median (IQR). Variables include demographic characteristics, blood pressure, laboratory values, health behaviors, medication use, and medical history

*IQR* interquartile range

### Incidence of primary outcomes according to CKM stage

During the follow-up, the incidence rates of all-cause mortality, cardiovascular death, and composite renal outcomes were 0.601%, 0.130%, and 0.246%, respectively. The highest frequencies of all-cause death and renal events were observed in stage 3 with 189 deaths (1.33%) and 180 renal events (1.27%). The incidence of cardiovascular death was comparable between stage 3 (0.331%) and stage 4a (0.363%) (Table 3).

**Table 3:** The number and frequency of occurrence of each outcome according to the CKM stage

CKM stage	Stage 0	Stage 1	Stage 2	Stage 3	Stage 4a	All
	(N = 29,262)	(N = 25,441)	(N = 175,589)	(N = 14,213)	(N = 21,751)	(N = 266,256)
All-cause mortality, N (%)	120 (0.410%)	86 (0.338%)	976 (0.556%)	189 (1.330%)	229 (1.053%)	1600 (0.601%)
Cardiovascular death, N (%)	21 (0.072%)	18 (0.071%)	181 (0.103%)	47 (0.331%)	79 (0.363%)	346 (0.130%)
Composite renal outcome, N (%)	34 (0.116%)	40 (0.157%)	299 (0.170%)	180 (1.266%)	101 (0.464%)	654 (0.246%)

This table presents the number and percentage of participants who experienced all-cause mortality, cardiovascular death, or the composite renal outcome within each CKM stage. The composite renal outcome was defined as progression to end-stage kidney disease (eGFR < 15 mL/min/1.73 m^2^), ≥ 40% decline in eGFR, or doubling of serum creatinine

### Associations between CKM stage and clinical outcomes

Multivariable Cox proportional hazards models were used to examine the associations between CKM stage and each clinical outcome (Table 4). Compared with stage 0, stages 1 and 2 did not significantly increase the HRs for mortality. For all-cause mortality, the HRs progressively increased with advancing stage, peaking at stage 4a (HR 1.79, 95% CI 1.41–2.28). A similar trend was observed for cardiovascular death, with the highest risk observed in stage 4a (HR 3.16, 95% CI 1.92–5.20). In contrast, the strongest association with the composite renal outcome was observed in stage 3 (HR 15.29, 95% CI 10.13–23.08), followed by stage 4a (HR 4.44, 95% CI 2.93–6.74). These findings indicated that while advanced CKM stages, particularly stage 4a, are the most predictive of mortality outcomes, stage 3, which is defined as severe CKD without overt CVD, represents the greatest risk of renal deterioration. Furthermore, analyses were conducted by age, gender, and CKD status. The results were presented in supplemental Fig. 1–2 and Supplemental Table 3.

**Table 4:** Hazard ratios for each primary outcome stratified by stage of CKM syndrome

	CKM stage	HR	95% CI	P value
All-cause death	Stage 0	References		
	Stage 1	0.88	(0.66–1.18)	0.399
	Stage 2	1.12	(0.91–1.38)	0.269
	Stage 3	1.71	(1.32–2.22)	< 0.001*
	Stage 4a	1.79	(1.41–2.28)	< 0.001*
CV death	Stage 0	References		
	Stage 1	0.86	(0.44–1.67)	0.656
	Stage 2	1.07	(0.67–1.69)	0.784
	Stage 3	1.92	(1.09–3.38)	0.025*
	Stage 4a	3.16	(1.92–5.20)	< 0.001*
Composite renal outcome	Stage 0	References		
	Stage 1	1.49	(0.93–2.39)	0.098
	Stage 2	1.69	(1.16–2.46)	0.006*
	Stage 3	15.29	(10.13–23.08)	< 0.001*
	Stage 4a	4.44	(2.93–6.74)	< 0.001*

Hazard ratios (HRs) and 95% confidence intervals (CIs) were estimated using multivariable Cox proportional hazards models, with CKM stage 0 as the reference. The models were adjusted for age, sex, smoking status, alcohol consumption, and exercise habits

*HR* hazard ratio, *CI* confidence interval

### Number of metabolic risk factors and outcomes

We examined the association between five metabolic risk factors (hypertension, dyslipidemia, diabetes, metabolic syndrome, and mild-to-moderate CKD) and clinical outcomes (Table 5A). HRs increased as the number of risk factors increased. The risks of all-cause mortality and composite renal outcomes increased significantly with ≥ 3 factors, while cardiovascular death increased with ≥ 4 factors. The highest risks were observed in participants with all five factors (all-cause mortality: HR 2.05, 95% CI 1.37–3.07; cardiovascular death: HR 2.55, 95% CI 1.16–5.61; composite renal outcome: HR 14.29, 95% CI 9.60–21.26). Notably, the renal risk increased synergistically with the number of accumulated factors.

**Table 5:** Associations between metabolic component risk factors and clinical outcomes

(A) stratification by number of the metabolic component risk factors				
	Number of component factor	HR	95% CI	P value
All-cause death	0 components	References		
	1 component	1.10	(0.91–1.34)	0.326
	2 components	1.20	(0.99–1.47)	0.068
	3 components	1.38	1.12–1.69)	0.002*
	4 components	1.61	(1.27–2.05)	< 0.001*
	5 components	2.05	(1.37–3.07)	< 0.001*
CV death	0 components	References		
	1 component	0.88	(0.57–1.37)	0.576
	2 components	1.26	(0.82–1.93)	0.286
	3 components	1.49	(0.96–2.31)	0.076
	4 components	1.91	(1.17–3.13)	0.010*
	5 components	2.55	(1.16–5.61)	0.020*
Composite renal outcome	0 components	References		
	1 components	1.06	(0.76–1.48)	0.740
	2 components	1.25	(0.89–1.75)	0.198
	3 components	2.26	(1.63–3.14)	< 0.001*
	4 components	4.54	(3.22–6.39)	< 0.001*
	5 components	14.29	(9.60–21.26)	< 0.001*
(B) stratification by type of the metabolic component risk factors				
	Type of component factor	HR	95% CI	P value
All-cause death	Hypertension	1.25	(1.10–1.43)	< 0.001*
	Dyslipidemia	0.80	(0.71–0.90)	< 0.001*
	Metabolic syndrome	1.31	(1.14–1.50)	< 0.001*
	Diabetes	1.37	(1.15–1.64)	< 0.001*
	Mild-moderate CKD	1.13	(1.01–1.27)	0.038*
CV death	Hypertension	1.88	(1.39–2.55)	< 0.001*
	Dyslipidemia	0.82	(0.63–1.05)	0.113
	Metabolic syndrome	1.19	(0.89–1.59)	0.237
	Diabetes	1.50	(1.04–2.17)	0.030*
	Mild-moderate CKD	1.18	(0.92–1.52)	0.192
Composite renal outcome	Hypertension	1.63	(1.33–2.01)	< 0.001*
	Dyslipidemia	1.14	(0.95–1.36)	0.164
	Metabolic syndrome	1.30	(1.07–1.57)	0.009*
	Diabetes	4.01	(3.27–4.91)	< 0.001*
	Mild-moderate CKD	0.84	(0.70–1.02)	0.086

Panels A and B present HRs and 95% CIs estimated using multivariable Cox proportional hazards models. Panel A shows associations according to the number of metabolic component factors. Panel B shows associations according to the type of component factors (hypertension, dyslipidemia, metabolic syndrome, diabetes, and mild-to-moderate CKD). The models were adjusted for age, sex, smoking status, alcohol consumption, and exercise habits

*HR* hazard ratio, *CI* confidence interval

### Impact of individual risk factors on outcomes

Hazard ratios were calculated to evaluate the contribution of each metabolic risk factor to the primary outcomes (Table 5B). For all-cause mortality, all metabolic dysfunctions, except dyslipidemia, significantly increased the risk, whereas dyslipidemia was associated with a reduced risk. For cardiovascular death, hypertension and diabetes were metabolic factor significantly associated with increased risk. For the composite renal outcome, hypertension, metabolic syndrome, and diabetes were significant predictors, with diabetes conferring the strongest risk (HR 4.01, 95% CI 3.27–4.91). Interestingly, hypertension and diabetes were found to be a metabolic factor that contributed to all outcomes.

### Contribution of CKM components to outcomes.

Finally, we assessed whether each of the three principal CKM components contributed to the risk of the primary outcomes (Table 6). Across all outcomes, risks increased with the number of factors (severe CKD, prior CVD, and metabolic dysfunction). Significant associations were also observed for two or more factors for all-cause or cardiovascular mortality. When considered individually, severe CKD and prior CVD were consistently stronger predictors than metabolic dysfunction. For the composite renal outcome, severe CKD was particularly prominent (HR 18.76, 95% CI 15.42–22.83).

**Table 6:** Hazard ratios for each primary outcome according to the number and type of core CKM risk factors

(A) stratification by number of the core CKM risk factors				
	Number of core CKM factor	HR	95% CI	P value
All-cause death	0 factors	References		
	1 factor	1.16	(0.97–1.40)	0.107
	2 factors	1.79	(1.43–2.23)	< 0.001*
	3 factors	3.81	(2.40–6.07)	< 0.001*
CV death	0 factors	References		
	1 factor	1.01	(0.68–1.50)	0.968
	2 factors	2.75	(1.76–4.31)	< 0.001*
	3 factors	7.60	(3.55–16.28)	< 0.001*
Composite renal outcome	0 factors	References		
	1 factor	1.37	(1.00–1.87)	0.049*
	2 factors	5.43	(3.87–7.62)	< 0.001*
	3 factors	35.07	(22.54–54.58)	< 0.001*
(B) stratification by type of the core CKM risk factors				
	Type of core CKM factor	HR	95% CI	P value
All-cause death	CVD	1.51	(1.29–1.75)	< 0.001*
	Severe CKD	1.81	(1.39–2.36)	< 0.001*
	Metabolic dysfunction	1.20	(1.00–1.43)	0.047*
CV death	CVD	2.63	(1.99–3.48)	< 0.001*
	Severe CKD	2.68	(1.69–4.26)	< 0.001*
	Metabolic dysfunction	1.11	(0.76–1.65)	0.585
Composite renal outcome	CVD	1.41	(1.12–1.78)	0.004*
	Severe CKD	18.76	(15.42–22.83)	< 0.001*
	Metabolic dysfunction	1.37	(1.02–1.85)	0.040*

Panels A and B present HRs and 95% CIs from multivariable Cox proportional hazards models. Panel A shows associations according to the number of core CKM risk factors (CVD, severe CKD, metabolic dysfunction). Panel B shows associations according to the type of core factors. The models were adjusted for age, sex, smoking status, alcohol consumption, and exercise habits

*HR* hazard ratio, *CI* confidence interval

## Discussion

In this large-scale Japanese cohort study, we evaluated the prevalence of CKM syndrome and its association with all-cause mortality, cardiovascular death, and composite renal outcomes. CKM syndrome, newly proposed by the American heart association in 2023, integrates cardiovascular, kidney, and metabolic dysfunctions into a unified staging system that reflects their shared pathophysiology and aims to guide early clinical decision-making.

Previous Asian studies have reported that stage 2 is the most common CKM stage. In South Korea, 51.6% of individuals were classified as stage 2, and a similar pattern was observed in China, where stage 2 accounted for 42.0% of the population [16, 17]. To the best of our knowledge, this is the first study to report the prevalence of CKM in a Japanese population. Stage 2 was the most common stage in our cohort (65.0%), a proportion notably higher than that in previous reports, possibly reflecting demographic differences, methodological factors, or unique features of the Japanese healthcare system. The age-related increase in advanced CKM stages was consistent with prior studies [18], emphasizing the importance of early detection and prevention in the Japanese population.

Beyond prevalence, this study clarified the prognostic significance of CKM staging. Consistent with previous reports, stage 4a conferred the highest risks of all-cause and cardiovascular mortality, whereas stage 3 showed the greatest risk for renal outcomes. To assess the contribution of CKD, stage 4a participants were stratified by CKD status. Those with current CKD had higher risks for all primary outcomes than those without CKD; however, their renal risk remained lower than that of stage 3 (Supplemental Fig. 2). Subgroup analyses stratified by eGFR stage showed a similar pattern. Although the significance of HRs within the same CKD stage varied, the risk of death generally tended to be higher in CKM stage 4a than stage 3. In contrast, the risk of renal outcomes tended to be higher in CKM stage 3 (Supplemental Table 3). This stage-specific heterogeneity underscores the clinical utility of CKM staging—stage 4a primarily predicts fatal outcomes, while stage 3 identifies individuals most vulnerable to rapid renal decline.

CKM stage 3 is often accompanied by proteinuria, mineral bone disorders, anemia, and structural kidney damage. These CKD-related abnormalities are established drivers of CVD through mechanisms such as uremia, inflammation, and oxidative stress [19]. Prior studies have shown that even modest albuminuria confers marked risks of mortality, CVD, and progression to ESKD [20–22]. Our findings suggest that Stage 3 represents a pivotal inflection point at which renal vulnerability and metabolic stress converge, thereby accelerating both eGFR decline and renal outcomes. While each metabolic dysfunction is individually recognized as a risk factor for kidney failure [23, 24], we observed that the accumulation of multiple metabolic risk factors amplified renal risk in a nonlinear fashion, suggesting synergistic effects.

One notable and paradoxical finding in this cohort was that the HRs between those with a history of CVD and those with severe CKD were almost the same, although the HRs for composite renal outcomes were significantly different. The HRs for all adverse outcomes were higher in CKM stage 4a with CKD than without CKD, leaving no doubt that CKD itself is an important risk factor. Further investigations revealed that patients with CVD had better control of BP and glucose (Supplemental Table 7). These findings suggest that patients with stage 4 who undergo more intensive medical monitoring and begin renoprotective therapy earlier may potentially delay death and CKD progression. Alternatively, the higher competing risk of death in this group may have contributed to the lower incidence of adverse renal outcomes. These findings highlight the importance of considering competing risks in research on CKM.

Although CKM stages 0–2 were not associated with adverse outcomes in our cohort, this finding differs from prior Chinese data showing a graded increase in mortality beginning at stage 2 [25]. Such discrepancies may reflect population differences or methodological variations. Indeed, stage 2 in our cohort represents a heterogeneous group with varying degrees of metabolic impairment. Although metabolic dysfunction at this stage did not independently elevate mortality or renal risk, the accumulation of CKM-defining comorbidities was strongly associated with progression to stages 3–4 (Supplemental Table 6). This pattern supports the concept of CKM as a progressive continuum: metabolic abnormalities act as permissive upstream drivers that increase physiological vulnerability, creating conditions under which severe CKD or overt CVD can subsequently develop. Once these downstream complications arise, they rapidly become the dominant determinants of mortality and renal decline, as demonstrated by the markedly elevated HRs in stages 3 and 4a. Therefore, the clinical relevance of stage 2 lies not in its immediate risk profile but in its role as a transitional state that enables disease escalation. This underscores the importance of early detection and aggressive management of metabolic dysfunction to prevent transition from stage 2 to the high-risk stages where prognosis worsens substantially.

This study had several limitations. First, the eGFR was calculated based on single annual measurements, which may have introduced biological variability. In other words, CKD classification was based on a single measurement, which differs from the standard requirement of persistent abnormalities. Although the outcome definitions adhered to the established criteria, misclassification due to fluctuations in serum creatinine levels was possible. Second, our CKM staging was derived from health check-up data and lacked advanced clinical parameters such as NT-proBNP or cardiac imaging, potentially leading to the underestimation of certain stages, particularly stage 3. Third, the accuracy of self-reports of disease and medication cannot be confirmed by other means. All subjects with cardiac disease and cerebrovascular events were included, but there was no data available on peripheral arterial disease or atrial fibrillation, so they could not be examined. Thus, classification of CKM stage 4a may be inaccurate. Finally, the median follow-up period of 4.2 years may have been insufficient to capture long-term outcomes. CKM syndrome is a conceptual framework intended for long-term risk assessment, and a longer follow-up period is required to fully evaluate the cardiovascular and renal trajectories across all stages.

## Conclusion

This study demonstrates the clinical value of CKM syndrome as a framework for risk stratification in the Japanese population. Stage 4 identifies individuals at the greatest risk of mortality, whereas stage 3 represents the critical inflection point for renal deterioration. These findings highlight the stage-specific patterns inherent in CKM and the importance of early intervention in stages 0–2 and renal-focused management in stage 3. Our results also reinforce the recognition of CKM syndrome as a progressive, dynamic construct. Metabolic dysfunction acts as a permissive step for progression, and preventing transition from stage 2 to stages 3–4 is crucial for clinical management. Recognizing CKM as a cumulative disease spectrum underscores the need for integrated strategies that address both cardiometabolic and renal health, thereby improving long-term outcomes across the continuum of chronic disease.

## Supplementary Information

Below is the link to the electronic supplementary material.

Supplementary file1 (DOCX 339 KB)

## Data Availability

The data used in this study are not available publicly.

## References

[1] Ndumele CE, Neeland IJ, Tuttle KR, Chow SL, Mathew RO, Khan SS, et al. A synopsis of the evidence for the science and clinical management of cardiovascular-kidney-metabolic (CKM) syndrome: a scientific statement from the American heart association. Circulation. 2023;148:1636–64. 10.1161/CIR.0000000000001186. 37807920 10.1161/CIR.0000000000001186

[2] Ndumele CE, Rangaswami J, Chow SL, Neeland IJ, Tuttle KR, Khan SS, et al. Cardiovascular-kidney-metabolic health: a presidential advisory from the American heart association. Circulation. 2023;148:1606–35. 10.1161/CIR.0000000000001184. 37807924 10.1161/CIR.0000000000001184

[3] Roth GA, Mensah GA, Johnson CO, Addolorato G, Ammirati E, Baddour LM, et al. Global burden of cardiovascular diseases and risk factors, 1990–2019. J Am Coll Cardiol. 2020;76:2982–3021. 10.1016/j.jacc.2020.11.010. 33309175 10.1016/j.jacc.2020.11.010 PMC7755038

[4] Ueki K, Sasako T, Okazaki Y, Kato M, Okahata S, Katsuyama H, et al. Effect of an intensified multifactorial intervention on cardiovascular outcomes and mortality in type 2 diabetes (J-DOIT3): an open-label, randomised controlled trial. Lancet Diabetes Endocrinol. 2017;5:951–64. 10.1016/S2213-8587(17)30327-3. 29079252 10.1016/S2213-8587(17)30327-3

[5] Ueki K, Sasako T, Okazaki Y, Miyake K, Nangaku M, Ohashi Y, et al. Multifactorial intervention has a significant effect on diabetic kidney disease in patients with type 2 diabetes. Kidney Int. 2021;99:256–66. 10.1016/j.kint.2020.08.012. 32891604 10.1016/j.kint.2020.08.012

[6] Prospective studies collabouration. Body-mass index and cause-specific mortality in 900,000 adults: colLabourative analyses of 57 prospective studies. Lancet. 2009;373:1083–96. 10.1016/S0140-6736(09)60318-4. 19299006 10.1016/S0140-6736(09)60318-4 PMC2662372

[7] Herrington WG, Smith M, Bankhead C, Matsushita K, Stevens S, Holt T, et al. Body-mass index and risk of advanced chronic kidney disease: prospective analyses from a primary care cohort of 1.4 million adults in England. PLoS ONE. 2017;12:e0173515. 10.1371/journal.pone.0173515. 28273171 10.1371/journal.pone.0173515 PMC5342319

[8] Iseki K, Asahi K, Yamagata K, Fujimoto S, Tsuruya K, Narita I, et al. Mortality risk among screened subjects of the specific health check and guidance program in Japan 2008–2012. Clin Exp Nephrol. 2017;21:978–85. 10.1007/s10157-017-1392-y. 28258498 10.1007/s10157-017-1392-y

[9] Japanese society of nephrology office@ jsn. or. jp. Essential points from evidence-based clinical practice guideline for chronic kidney disease 2023. Clin Exp Nephrol. 2024;28(6):473–95. 10.1007/s10157-024-02497-4. 38713253 10.1007/s10157-024-02497-4 PMC11116248

[10] Matsuo S, Imai E, Horio M, Yasuda Y, Tomita K, Nitta K, et al. Revised equations for estimated GFR from serum creatinine in Japan. Am J Kidney Dis. 2009;53:982–92. 10.1053/j.ajkd.2008.12.034. 19339088 10.1053/j.ajkd.2008.12.034

[11] Diagnosis and classification of diabetes mellitus. Diabetes Care. 2011;34:S62–9. 10.2337/dc11-S062. 21193628 10.2337/dc11-S062 PMC3006051

[12] Umemura S, Arima H, Arima S, Asayama K, Dohi Y, Hirooka Y, et al. The Japanese society of hypertension guidelines for the management of hypertension (JSH 2019). Hypertens Res. 2019;42:1235–481. 10.1038/s41440-019-0284-9. 31375757 10.1038/s41440-019-0284-9

[13] Decreased GJ. Definition and classification of CKD. Kidney Int Suppl (2011). 2013;3:19–62. 10.1038/kisup.2012.64. 25018975 10.1038/kisup.2012.64 PMC4089693

[14] Khan SS, Matsushita K, Sang Y, Ballew SH, Grams ME, Surapaneni A, et al. Development and validation of the American heart association’s PREVENT equations. Circulation. 2024;149:430–49. 10.1161/CIRCULATIONAHA.123.067626. 37947085 10.1161/CIRCULATIONAHA.123.067626 PMC10910659

[15] Stevens PE, Ahmed SB, Carrero JJ, Foster B, Francis A, Hall RK, et al. KDIGO 2024 clinical practice guideline for the evaluation and management of chronic kidney disease. Kidney Int. 2024;105:S117–314. 10.1016/j.kint.2023.10.018. 38490803 10.1016/j.kint.2023.10.018

[16] Zhang H, Hu Z, Wu J, Li Y, Jiang S, Jin L, et al. Prevalence of cardiovascular-kidney-metabolic syndrome stages among middle-aged and older adults in China. JACC Asia. 2025;5:393–5. 10.1016/j.jacasi.2024.12.003. 40049933 10.1016/j.jacasi.2024.12.003 PMC11934037

[17] Hong S-B, Kim J-E, Han SS, Shearer JJ, Joo J, Choi J-Y, et al. Prevalence of cardiovascular-kidney-metabolic syndrome in Korea: Korea national health and nutrition examination survey 2011–2021. Epidemiol Health. 2025;47:e2025005. 10.4178/epih.e2025005. 39961594 10.4178/epih.e2025005 PMC12062855

[18] Tsai M-K, Kao JT-W, Wong C-S, Liao C-T, Lo W-C, Chien K-L, et al. Cardiovascular–kidney–metabolic syndrome and all-cause and cardiovascular mortality: a retrospective cohort study. PLoS Med. 2025;22(6):1004629. 10.1371/journal.pmed.1004629 PMC12200875 40570007

[19] Sarnak MJ, Amann K, Bangalore S, Cavalcante JL, Charytan DM, Craig JC, et al. Chronic kidney disease and coronary artery disease. J Am Coll Cardiol. 2019;74:1823–38. 10.1016/j.jacc.2019.08.1017. 31582143 10.1016/j.jacc.2019.08.1017

[20] Astor BC, Matsushita K, Gansevoort RT, van der Velde M, Woodward M, Levey AS, et al. Lower estimated glomerular filtration rate and higher albuminuria are associated with mortality and end-stage renal disease: a collaborative meta-analysis of kidney disease population cohorts. Kidney Int. 2011;79:1331–40. 10.1038/ki.2010.550. 21289598 10.1038/ki.2010.550 PMC3917543

[21] Gansevoort RT, Matsushita K, van der Velde M, Astor BC, Woodward M, Levey AS, et al. Lower estimated GFR and higher albuminuria are associated with adverse kidney outcomes: a collaborative meta-analysis of general and high-risk population cohorts. Kidney Int. 2011;80:93–104. 10.1038/ki.2010.531. 21289597 10.1038/ki.2010.531 PMC3959732

[22] Matsushita K, Coresh J, Sang Y, Chalmers J, Fox C, Guallar E, et al. Estimated glomerular filtration rate and albuminuria for prediction of cardiovascular outcomes: a collaborative meta-analysis of individual participant data. Lancet Diabetes Endocrinol. 2015;3:514–25. 10.1016/S2213-8587(15)00040-6. 26028594 10.1016/S2213-8587(15)00040-6 PMC4594193

[23] Go AS, Chertow GM, Fan D, McCulloch CE, Hsu C. Chronic kidney disease and the risks of death, cardiovascular events, and hospitalization. N Engl J Med. 2004;351:1296–305. 10.1056/NEJMoa041031. 15385656 10.1056/NEJMoa041031

[24] Zhou Y, Liu Y, Wu L, Zhang Y, Wen H, Hu J, et al. Causal insights into major risk factors for diabetic kidney disease: a comprehensive meta-analysis and Mendelian randomization study. Ren Fail. 2025;47:1–10. 10.1080/0886022X.2025.2468741. 10.1080/0886022X.2025.2468741 PMC11984328 40012233

[25] Zheng C, Cai A, Sun M, Wang X, Song Q, Pei X, et al. Prevalence and mortality of cardiovascular-kidney-metabolic syndrome in China. JACC Asia. 2025;5:898–910. 10.1016/j.jacasi.2025.04.007. 40498431 10.1016/j.jacasi.2025.04.007 PMC12277190

