# Supplementary material for: Stage-specific risks of mortality and renal outcomes in cardiovascular-kidney-metabolic syndrome: findings from a nationwide Japanese cohort
Source: Clin Exp Nephrol. 2025 Dec 17;30(3):434–45. doi: 10.1007/s10157-025-02800-x (PMC12950010; doi:10.1007/s10157-025-02800-x)
Supplement: Supplementary file 1 — Supplementary file1 (DOCX 339 KB) [file 10157_2025_2800_MOESM1_ESM.docx]

(**Supplemental Tables + Supplemental Table legends**).

| **CKM stage** | **Stage 0** | | **Stage 1** | | **Stage 2** | | **Stage 3** | | **Stage 4a** | | **Stage 4b** | | **Total** |
| --- | --- | --- | --- | --- | --- | --- | --- | --- | --- | --- | --- | --- | --- |
| **40-44** | 7029 | (27.22%) | 3997 | (15.48%) | 14269 | (55.27%) | 87 | (0.34%) | 437 | (1.69%) | 0 | (0.00%) | 25819 |
| **45-49** | 4606 | (21.04%) | 2926 | (13.37%) | 13604 | (62.14%) | 160 | (0.73%) | 594 | (2.71%) | 1 | (0.00%) | 21891 |
| **50-54** | 4284 | (15.19%) | 3243 | (11.50%) | 19243 | (68.24%) | 324 | (1.15%) | 1098 | (3.89%) | 8 | (0.03%) | 28200 |
| **55-59** | 5626 | (12.45%) | 4773 | (10.56%) | 31618 | (69.95%) | 793 | (1.75%) | 2388 | (5.28%) | 5 | (0.01%) | 45203 |
| **60-64** | 7306 | (9.57%) | 7246 | (9.49%) | 53654 | (70.27%) | 2271 | (2.97%) | 5864 | (7.68%) | 9 | (0.01%) | 76350 |
| **65-69** | 7487 | (7.68%) | 7693 | (7.89%) | 66593 | (68.34%) | 5578 | (5.72%) | 10075 | (10.34%) | 21 | (0.02%) | 97447 |
| **70-74** | 5046 | (5.51%) | 5410 | (5.90%) | 52423 | (57.20%) | 16186 | (17.66%) | 12545 | (13.69%) | 31 | (0.03%) | 91641 |
| **Total** | 41384 | (10.71%) | 35288 | (9.13%) | 251404 | (65.04%) | 25399 | (6.57%) | 33001 | (8.54%) | 75 | (0.02%) | 386551 |

**Supplemental Table 1.　 Age-stratified participants (A) and prevalence of each CKM stage (B) in Japan (Analysis 1)**

Percentages represent the proportion of each stage within each age category in Figure 2.

|  |  | **CKM stage** | **HR** |  |  | **95%CI** | | |  |  | **P value** |  |
| --- | --- | --- | --- | --- | --- | --- | --- | --- | --- | --- | --- | --- |
|  | All-cause death | stage 0 | Reference |  |  |  |  |  |  |  |  |  |
|  |  | stage 1 | 0.83 |  | ( | 0.63 | - | 1.09 | ) |  | 0.185 |  |
|  |  | stage 2 | 1.42 |  | ( | 1.18 | - | 1.72 | ) |  | <0.001* |  |
|  |  | stage 3 | 4.46 |  | ( | 3.55 | - | 5.61 | ) |  | <0.001* |  |
|  |  | stage 4a | 2.89 |  | ( | 2.32 | - | 3.61 | ) |  | <0.001* |  |
|  | CV death | stage 0 | Reference |  |  |  |  |  |  |  |  |  |
|  |  | stage 1 | 0.99 |  | ( | 0.53 | - | 1.86 | ) |  | 0.980 |  |
|  |  | stage 2 | 1.50 |  | ( | 0.96 | - | 2.36 | ) |  | 0.078 |  |
|  |  | stage 3 | 6.11 |  | ( | 3.65 | - | 10.22 | ) |  | <0.001* |  |
|  |  | stage 4a | 5.63 |  | ( | 3.48 | - | 9.11 | ) |  | <0.001* |  |
|  | Composite renal outcome | stage 0 | Reference |  |  |  |  |  |  |  |  |  |
|  |  | stage 1 | 1.40 |  | ( | 0.89 | - | 2.21 | ) |  | 0.149 |  |
|  |  | stage 2 | 1.58 |  | ( | 1.11 | - | 2.26 | ) |  | 0.011* |  |
|  |  | stage 3 | 14.99 |  | ( | 10.38 | - | 21.65 | ) |  | <0.001* |  |
|  |  | stage 4a | 4.52 |  | ( | 3.06 | - | 6.66 | ) |  | <0.001* |  |

**Supplemental Table 2. Unadjusted hazard ratios for each primary outcome stratified by CKM stage**

This table presents the unadjusted hazard ratios (HRs) with 95% confidence intervals (CIs), using CKM stage 0 as the reference group. These results correspond to the unadjusted analyses for comparison with the adjusted models presented in Table 4. HR, hazard ratio; CI, confidence interval.

|  |  | **stage 0** | **stage 1** | **stage 2** | **stage 3** | **stage 4a** |
| --- | --- | --- | --- | --- | --- | --- |
| All-cause death | **CKD G1** | 0.65 (0.32-1.31) | 0.55 (0.26-1.17) | 0.77 (0.42-1.40) | Reference | 1.15 (0.58-2.30) |
|  | **CKD G2** | 0.63 (0.45-0.89) | 0.58 (0.4-0.83) | 0.70 (0.53-0.92) | Reference | 1.05 (0.77-1.43) |
|  | **CKD G3a** | - | - | 0.64 (0.45-0.91) | Reference | 1.13 (0.75-1.71) |
|  | **CKD G3b** | - | - | 0.64 (0.29-1.44) | Reference | 1.17 (0.56-2.45) |
|  | **CKD G4** | - | - | - | Reference | 3.16 (0.8-12.48) |
| CV death | **CKD G1** | 1.17 (0.12-11.24) | 0.75 (0.06-8.91) | 1.46 (0.19-11.48) | Reference | 4.31 (0.51-36.34) |
|  | **CKD G2** | 0.74 (0.35-1.58) | 0.69 (0.31-1.53) | 0.81 (0.44-1.49) | Reference | 2.03 (1.06-3.87) |
|  | **CKD G3a** | - | - | 0.30 (0.15-0.62) | Reference | 1.27 (0.62-2.57) |
|  | **CKD G3b** | - | - | *Not estimable* | Reference | 3.13 (0.81-12.15) |
|  | **CKD G4** | - | - | - | Reference | 5.35 (0.18-158.65) |
| Composite renal outcome | **CKD G1** | 0.17 (0.08-0.35) | 0.18 (0.09-0.38) | 0.26 (0.15-0.45) | Reference | 0.34 (0.17-0.69) |
|  | **CKD G2** | 0.19 (0.09-0.39) | 0.36 (0.19-0.68) | 0.35 (0.21-0.58) | Reference | 0.60 (0.34-1.06) |
|  | **CKD G3a** | - | - | 0.09 (0.05-0.18) | Reference | 0.42 (0.2-0.85) |
|  | **CKD G3b** | - | - | 0.03 (0.00-0.20) | Reference | 0.66 (0.33-1.31) |
|  | **CKD G4** | - | - | - | Reference | 1.37 (0.82-2.28) |

**Supplemental Table 3. Adjusted hazard ratios for all-cause death, cardiovascular disease, and composite renal outcome according to CKM stage, stratified by CKD stage.**

Cox proportional hazards models were used to estimate adjusted hazard ratios (HRs) and 95% confidence intervals (CIs) for each outcome, stratified by CKD stage (G1–G4). No patients met the criteria for CKDG5. For each CKD stage, CKM stage 3 was used as the reference category. “Not estimable” indicates that the number of events was too small to calculate a reliable estimate. The models were adjusted for age, sex, smoking status, alcohol consumption, and exercise habits.

| **(A) stratification by number of the metabolic component risk factors (unadjusted)** | | | | | | | | | | | | |
| --- | --- | --- | --- | --- | --- | --- | --- | --- | --- | --- | --- | --- |
|  |  | **Number of component factor** | **HR** |  |  | **95%CI** | | |  |  | **P value** |  |
|  | All-cause death | 0 components | Reference |  |  |  |  |  |  |  |  |  |
|  |  | 1 component | 1.45 |  | ( | 1.21 | - | 1.74 | ) |  | <0.001* |  |
|  |  | 2 components | 1.65 |  | ( | 1.37 | - | 1.98 | ) |  | <0.001* |  |
|  |  | 3 components | 1.94 |  | ( | 1.60 | - | 2.35 | ) |  | <0.001* |  |
|  |  | 4 components | 2.46 |  | ( | 1.97 | - | 3.07 | ) |  | <0.001* |  |
|  |  | 5 components | 4.14 |  | ( | 2.92 | - | 5.86 | ) |  | <0.001* |  |
|  | CV death | 0 components | Reference |  |  |  |  |  |  |  |  |  |
|  |  | 1 component | 1.22 |  | ( | 0.81 | - | 1.85 | ) |  | 0.343 |  |
|  |  | 2 components | 1.88 |  | ( | 1.25 | - | 2.81 | ) |  | 0.002* |  |
|  |  | 3 components | 2.17 |  | ( | 1.43 | - | 3.29 | ) |  | <0.001* |  |
|  |  | 4 components | 3.35 |  | ( | 2.12 | - | 5.29 | ) |  | <0.001* |  |
|  |  | 5 components | 6.56 |  | ( | 3.43 | - | 12.55 | ) |  | <0.001* |  |
|  | Composite renal outcome | 0 components | Reference |  |  |  |  |  |  |  |  |  |
|  |  | 1 component | 1.12 |  | ( | 0.81 | - | 1.53 | ) |  | 0.497 |  |
|  |  | 2 components | 1.35 |  | ( | 0.98 | - | 1.85 | ) |  | 0.063 |  |
|  |  | 3 components | 2.57 |  | ( | 1.89 | - | 3.50 | ) |  | <0.001* |  |
|  |  | 4 components | 5.00 |  | ( | 3.63 | - | 6.89 | ) |  | <0.001* |  |
|  |  | 5 components | 16.92 |  | ( | 11.67 | - | 24.52 | ) |  | <0.001* |  |
|  |  |  |  |  |  |  |  |  |  |  |  |  |
| **(B) stratification by type of the metabolic component risk factors (unadjusted)** | | | | | | | | | | | | |
|  |  | **Type of component factor** | **HR** |  |  | **95%CI** | | |  |  | **P value** |  |
|  | All-cause death | Hypertension | 1.57 |  | ( | 1.39 | - | 1.76 | ) |  | <0.001* |  |
|  |  | Dyslipidemia | 0.80 |  | ( | 0.72 | - | 0.89 | ) |  | <0.001* |  |
|  |  | Metabolic syndrome | 1.17 |  | ( | 1.03 | - | 1.32 | ) |  | 0.016* |  |
|  |  | Diabetes | 1.79 |  | ( | 1.52 | - | 2.11 | ) |  | <0.001* |  |
|  |  | Mild-moderate CKD | 1.37 |  | ( | 1.23 | - | 1.52 | ) |  | <0.001* |  |
|  | CV death | Hypertension | 2.38 |  | ( | 1.80 | - | 3.16 | ) |  | <0.001* |  |
|  |  | Dyslipidemia | 0.85 |  | ( | 0.68 | - | 1.07 | ) |  | 0.166 |  |
|  |  | Metabolic syndrome | 1.08 |  | ( | 0.83 | - | 1.40 | ) |  | 0.576 |  |
|  |  | Diabetes | 2.06 |  | ( | 1.49 | - | 2.85 | ) |  | <0.001* |  |
|  |  | Mild-moderate CKD | 1.46 |  | ( | 1.16 | - | 1.83 | ) |  | 0.001* |  |
|  | Composite renal outcome | Hypertension | 1.66 |  | ( | 1.36 | - | 2.02 | ) |  | <0.001* |  |
|  |  | Dyslipidemia | 1.18 |  | ( | 0.99 | - | 1.40 | ) |  | 0.062 |  |
|  |  | Metabolic syndrome | 1.25 |  | ( | 1.04 | - | 1.51 | ) |  | 0.017* |  |
|  |  | Diabetes | 4.30 |  | ( | 3.55 | - | 5.21 | ) |  | <0.001* |  |
|  |  | Mild-moderate CKD | 0.88 |  | ( | 0.73 | - | 1.06 | ) |  | 0.176 |  |

**Supplemental Table 4. Unadjusted hazard ratios for each primary outcome stratified by number and type of component factors**

This table presents unadjusted hazard ratios (HRs) with 95% confidence intervals (CIs). Reference groups were defined as (A) participants with no component factors (number analysis) and (B) participants without each specific disease (type analysis). These results correspond to the unadjusted analyses for comparison with the adjusted models presented in Table 5. HR, hazard ratio; CI, confidence interval.

| **(A) stratification by number of the core CKM risk factors (unadjusted)** | | | | | | | | | | | | |
| --- | --- | --- | --- | --- | --- | --- | --- | --- | --- | --- | --- | --- |
|  |  | **Number of core CKM factor** | **HR** |  |  | **95%CI** | | |  |  | **P value** |  |
|  | All-cause death | 0 factors | Reference |  |  |  |  |  |  |  |  |  |
|  |  | 1 factor | 1.56 |  | ( | 1.31 | - | 1.85 | ) |  | <0.001* |  |
|  |  | 2 factors | 3.03 |  | ( | 2.47 | - | 3.71 | ) |  | <0.001* |  |
|  |  | 3 factors | 9.35 |  | ( | 6.21 | - | 14.09 | ) |  | <0.001* |  |
|  | CV death | 0 factors | Reference |  |  |  |  |  |  |  |  |  |
|  |  | 1 factor | 1.45 |  | ( | 0.99 | - | 2.12 | ) |  | 0.057 |  |
|  |  | 2 factors | 4.97 |  | ( | 3.27 | - | 7.55 | ) |  | <0.001* |  |
|  |  | 3 factors | 18.35 |  | ( | 9.19 | - | 36.63 | ) |  | <0.001* |  |
|  | Composite Renal Outcome | 0 factors | Reference |  |  |  |  |  |  |  |  |  |
|  |  | 1 factor | 1.41 |  | ( | 1.05 | - | 1.89 | ) |  | 0.021* |  |
|  |  | 2 factors | 6.66 |  | ( | 4.88 | - | 9.08 | ) |  | <0.001* |  |
|  |  | 3 factors | 40.53 |  | ( | 26.78 | - | 61.35 | ) |  | <0.001* |  |
|  |  |  |  |  |  |  |  |  |  |  |  |  |
| **(B) stratification by type of the core CKM risk factors (unadjusted)** | | | | | | | | | | | | |
|  |  | **Type of core CKM factor** | **HR** |  |  | **95%CI** | | |  |  | **P value** |  |
|  | All-cause death | CVD | 1.87 |  | ( | 1.63 | - | 2.16 | ) |  | <0.001* |  |
|  |  | severe CKD | 2.57 |  | ( | 2.04 | - | 3.26 | ) |  | <0.001* |  |
|  |  | metabolic dysfunction | 1.61 |  | ( | 1.36 | - | 1.90 | ) |  | <0.001* |  |
|  | CV death | CVD | 3.19 |  | ( | 2.47 | - | 4.11 | ) |  | <0.001* |  |
|  |  | severe CKD | 3.88 |  | ( | 2.59 | - | 5.81 | ) |  | <0.001* |  |
|  |  | metabolic dysfunction | 1.59 |  | ( | 1.10 | - | 2.31 | ) |  | 0.014* |  |
|  | Composite renal outcome | CVD | 1.58 |  | ( | 1.27 | - | 1.96 | ) |  | <0.001* |  |
|  |  | severe CKD | 20.14 |  | ( | 16.79 | - | 24.15 | ) |  | <0.001* |  |
|  |  | metabolic dysfunction | 1.40 |  | ( | 1.06 | - | 1.86 | ) |  | 0.019* |  |

**Supplemental Table 5. Unadjusted hazard ratios for each primary outcome according to the number and type of core CKM risk factors**

This table presents unadjusted hazard ratios (HRs) with 95% confidence intervals (CIs). Reference groups were defined as (A) participants with no component factors (number analysis) and (B) participants without each specific disease (type analysis). These results correspond to the unadjusted analyses for comparison with the adjusted models presented in Table 6. HR, hazard ratio; CI, confidence interval.

| **(A) stratification by number of the component factors (unadjusted)** | | | | | | |  |  |  |  |  |  |
| --- | --- | --- | --- | --- | --- | --- | --- | --- | --- | --- | --- | --- |
|  | | **Number of component factor** | | **HR** |  |  | **95%CI** | | |  |  | **P value** |
| CKM stage progression | | 0 components | | Reference |  |  |  |  |  |  |  |  |
|  | | 1 component | | 3.89 |  | ( | 3.51 | - | 4.30 | ) |  | <0.001* |
|  | | 2 components | | 7.66 |  | ( | 6.94 | - | 8.46 | ) |  | <0.001* |
|  | | 3 components | | 11.94 |  | ( | 10.82 | - | 13.19 | ) |  | <0.001* |
|  | | 4 components | | 20.54 |  | ( | 18.53 | - | 22.77 | ) |  | <0.001* |
|  | | 5 components | | 32.15 |  | ( | 27.62 | - | 37.42 | ) |  | <0.001* |
|  |  | |  |  | |  |  |  |  |  |  |  |
| **(B) stratification by type of the component factors (unadjusted)** | | | | | |  |  |  |  |  |  |  |
|  | | **Type of component factor** | | **HR** |  |  | **95%CI** | | |  |  | **P value** |
| CKM stage progression | | Hypertension | | 4.25 |  | ( | 4.05 | - | 4.47 | ) |  | <0.001* |
|  | | Dyslipidemia | | 1.02 |  | ( | 0.99 | - | 1.06 | ) |  | 0.209 |
|  | | Metabolic syndrome | | 1.36 |  | ( | 1.31 | - | 1.41 | ) |  | <0.001* |
|  | | Diabetes | | 2.40 |  | ( | 2.27 | - | 2.52 | ) |  | <0.001* |
|  | | Mild-moderate CKD | | 2.49 |  | ( | 2.41 | - | 2.57 | ) |  | <0.001* |
|  | | | | | | |  |  |  |  |  |  |
|  | | | | | | |  |  |  |  |  |  |
| **(C) stratification by number of the component factors** | | | | | | |  |  |  |  |  |  |
|  | | **Number of component factor** | | **HR** |  |  | **95%CI** | | |  |  | **P value** |
| CKM stage progression | | 0 components | | Reference |  |  |  |  |  |  |  |  |
|  | | 1 component | | 2.76 |  | ( | 2.48 | - | 3.07 | ) |  | <0.001* |
|  | | 2 components | | 5.03 |  | ( | 4.54 | - | 5.58 | ) |  | <0.001* |
|  | | 3 components | | 8.31 |  | ( | 7.49 | - | 9.23 | ) |  | <0.001* |
|  | | 4 components | | 13.86 |  | ( | 12.44 | - | 15.45 | ) |  | <0.001* |
|  | | 5 components | | 35.39 |  | ( | 30.10 | - | 41.60 | ) |  | <0.001* |
|  | |  | |  |  |  |  |  |  |  |  |  |
| **(D) stratification by type of the component factors** | | | | | |  |  |  |  |  |  |  |
|  | | **Type of component factor** | | **HR** |  |  | **95%CI** | | |  |  | **P value** |
| CKM stage progression | | Hypertension | | 3.19 |  | ( | 3.03 | - | 3.36 | ) |  | <0.001* |
|  | | Dyslipidemia | | 1.11 |  | ( | 1.07 | - | 1.16 | ) |  | <0.001* |
|  | | Metabolic syndrome | | 1.71 |  | ( | 1.64 | - | 1.78 | ) |  | <0.001* |
|  | | Diabetes | | 2.89 |  | ( | 2.74 | - | 3.05 | ) |  | <0.001* |
|  | | Mild-moderate CKD | | 1.89 |  | ( | 1.82 | - | 1.95 | ) |  | <0.001* |

**Supplemental Table 6. Hazard ratios for risk of progression to CKM stages 3–4 among patients with CKM stages 0–2**

Multivariable Cox proportional hazards models were used to estimate hazard ratios (HRs) and 95% confidence intervals (CIs) for progression from CKM stages 0–2 to 3–4. Analyses were stratified according to (A/C) the number of component factors and (B/D) the type of component factors. Panels A and B present unadjusted models, whereas panels C and D present adjusted models. The reference group consisted of patients with no component factors (A/C) or without each specific component factor (B/D). Adjusted models (C/D) included age, sex, smoking status, alcohol consumption, and exercise habits. HR, hazard ratio; CI, confidence interval.

| **Condition** | **History of CVD** | **Disease status** | **Subjects** | **Using medications** | **Treatment rate (%)** | **Control indicator, Median (IQR)** | | | | | |
| --- | --- | --- | --- | --- | --- | --- | --- | --- | --- | --- | --- |
| **Diabetes** | (−) | (−) | 9,367 | 0 | – | HbA1c  (%): | 5.30 | (5.1–5.6) | FBS (mg/dL): | 97 | (90-106) |
|  | (+) | (−) | 19,141 | 0 | – |  | 5.20 | (5.0–5.5) |  | 93 | (87-101) |
|  | (−) | (+) | 4,817 | 3,738 | 77.60 |  | 7.00 | (6.3–7.9) |  | 141 | (121-171) |
|  | (+) | (+) | 2,478 | 2,193 | 88.50 |  | 6.50 | (5.9–7.2) |  | 129 | (109-150) |
| **Hypertension** | (-) | (-) | 1045 | 0 | - | sBP (mmHg): | 118 | (109–124) | dBP (mmHg): | 70 | (62–72) |
|  | (+) | (-) | 4426 | 0 | - |  | 116 | (108–122) |  | 70 | (62–72) |
|  | (-) | (+) | 13168 | 9782 | 74.29 |  | 141 | (132–154) |  | 80 | (74–88) |
|  | (+) | (+) | 17324 | 12480 | 72.04 |  | 134 | (126–142) |  | 80 | (70–84) |

**Supplemental Table 7. Pharmacological interventions and clinical control of diabetes and hypertension in CKM stage 3–4 patients, stratified according to CVD history and disease status**

This table summarizes the medication use and clinical control for diabetes and hypertension in patients with CKM stages 3–4, stratified according to the presence or absence of cardiovascular disease (CVD) and comorbidities. For each condition, the table presents the number of patients, proportion of patients receiving disease-specific medications, and degree of control of the clinical parameters (HbA1c and fasting blood glucose for diabetes; systolic and diastolic blood pressure for hypertension). Patients with CVD tend to achieve better disease management than those without CVD.

Data are presented as numbers, percentages, or medians (IQR). IQR, interquartile range; FBS, fasting blood sugar; sBP, systolic blood pressure; dBP, diastolic blood pressure.


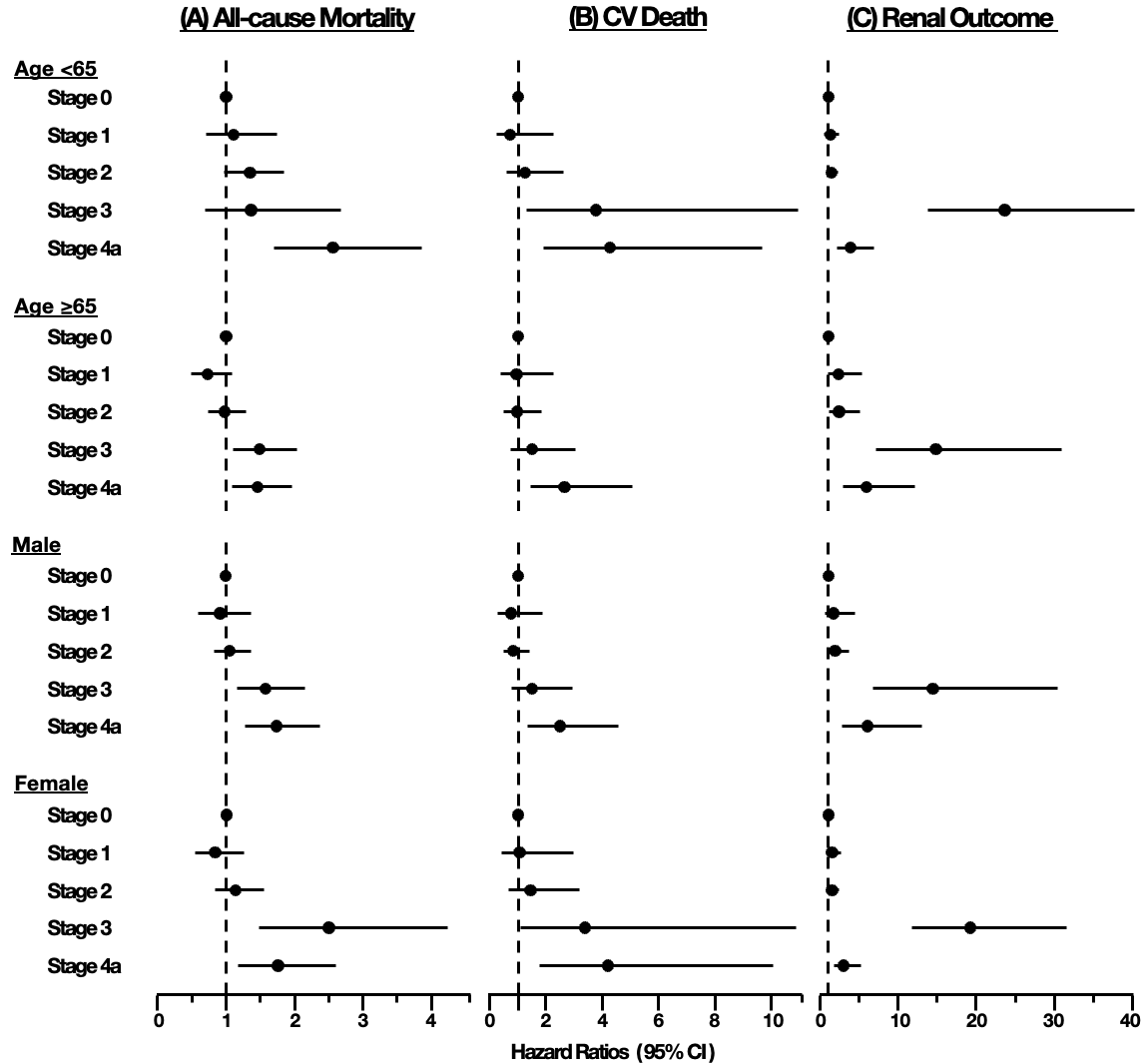


**Supplemental Figure 1. Subgroup analyses of the associations between CKM stage and (A) all-cause mortality, (B) cardiovascular death, and (C) renal outcomes.**

Hazard ratios (HRs) and 95% CIs were estimated using multivariable Cox models adjusted for age, sex, smoking status, alcohol consumption, and exercise habits. Subgroup analyses were performed by age (<65 vs. ≥65 years) and sex (male vs. female). CKM stage 0 was used as the reference category.


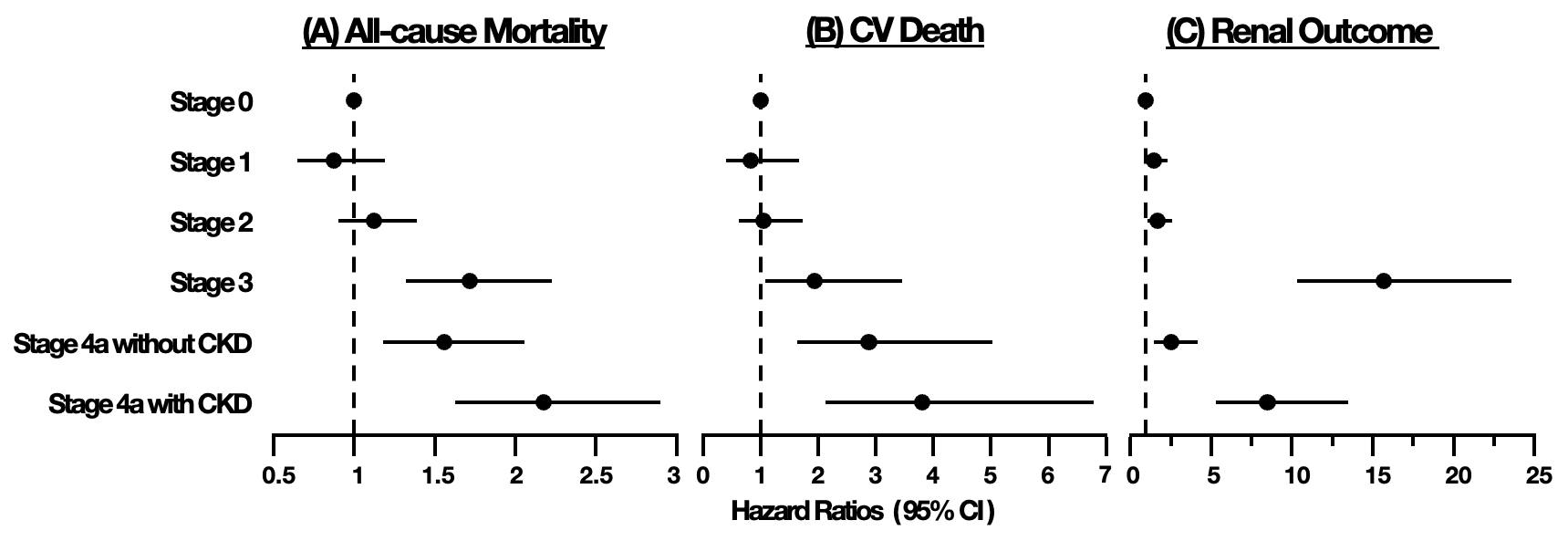


**Supplemental Figure 2. Hazard ratios for each primary outcome stratified according to the CKM stage, reflecting the CKD.**

Hazard ratios (HRs) were estimated using multivariable Cox models adjusted for age, sex, smoking status, alcohol consumption, and exercise habits. Participants with CKM stage 4a were further stratified by CKD status. CKM stage 0 served as the reference category.
